# Imaging the Anterior and Posterior Cruciate Ligaments

**DOI:** 10.5334/jbr-btr.1197

**Published:** 2016-11-19

**Authors:** Anagah P. Parkar

**Affiliations:** 1 Haraldsplass Deaconess Hospital, NO

**Keywords:** ACL, PCL, MRI, CT, Ruptures, Post-operative imaging

## Abstract

The anterior and posterior cruciate ligaments are important stabilizers of the knee joint function. Although they are both similar in their native appearance, they possess slightly different properties and complement each other’s function. The imaging findings differ between the anterior and posterior cruciate ligaments. While MRI is the main imaging modality, radiographs and CT have a role in pre- and post-operative imaging. The aim of this review is to present pre-and post-operative imaging findings of injured cruciate ligaments. A special emphasis will be placed on the potential pitfalls in cruciate ligament imaging.

## Anterior Cruciate Ligament (ACL) Imaging

The anterior cruciate ligament (ACL) courses from the lateral femoral condyle to the anterior mid portion of the tibia, attaching just anterior to the tibial spine. In the femur and tibia the attachments spread out like fans or ducks foot [1,2,3]. Anatomically it is two bundled but there is an ongoing debate whether the ACL is functionally two bundled or not [4].

Injury to the ACL usually occurs due to extreme hyper-extension or a twisting trauma. A severe injury may leave the knee functionally ACL-deficient.

**Figure 1:**
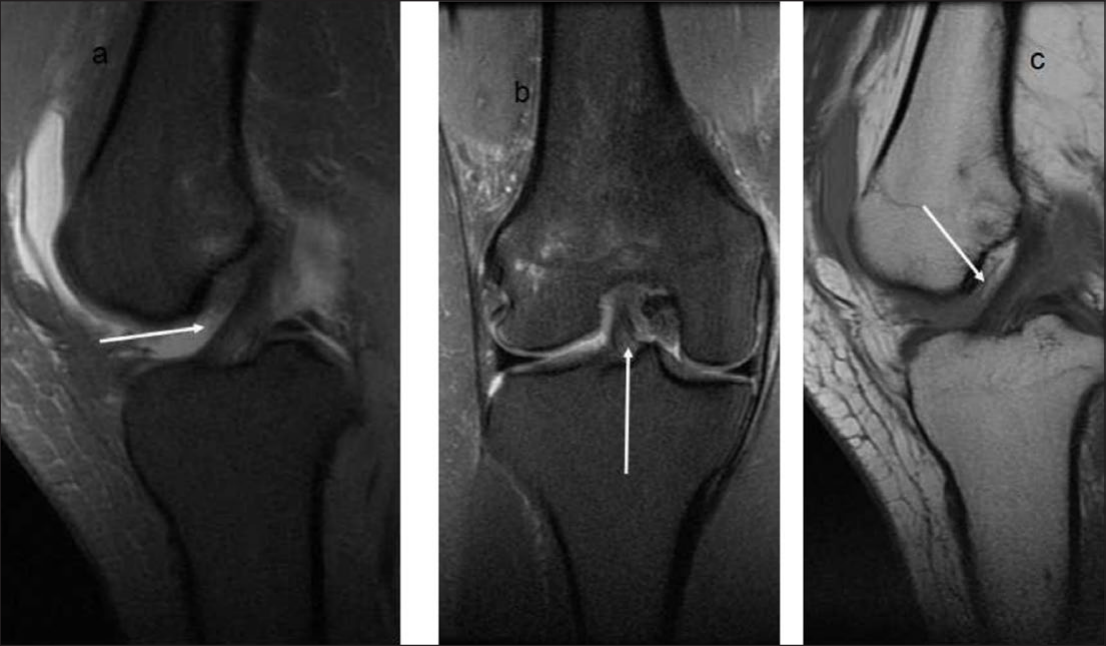
**(a)** Sagittal and **(b)** coronal T2- and sagittal T1-weighted images. **(c)** The ACL is dark on all pulse sequences (*arrows*).

**Figure 2:**
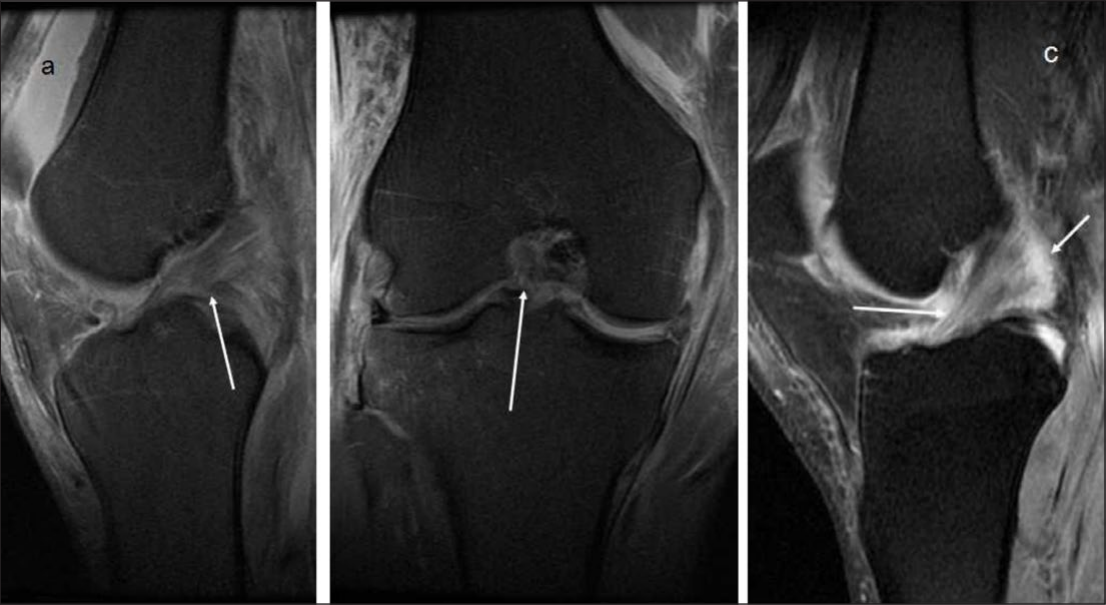
**(a)** Sagittal and **(b)** coronal T2-weighted MRI. High signal in a thickened ACL, but no discontinuity in the fibres, consistent with partial rupture. **(c)** Different patient, high signal in the ACL with lot of fluid around it, but intact fibres anteriorly (*arrows*).

**Figure 3:**
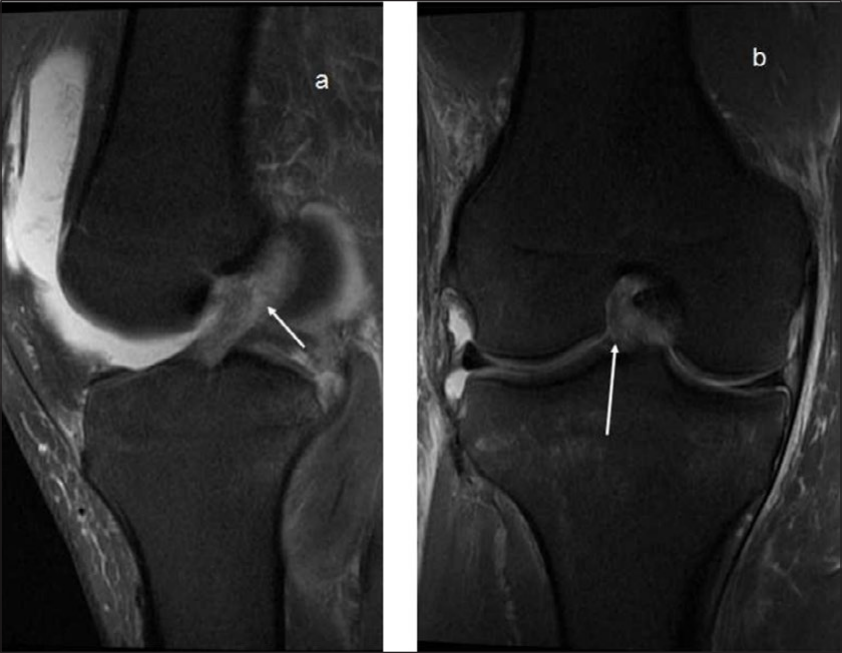
Sagittal and coronal T2-weighted MRI High signal in the ACL, but with discontinuity in the fibres in the posterior part, but still intact anteriorly, consistent with a partial rupture.

**Figure 4:**
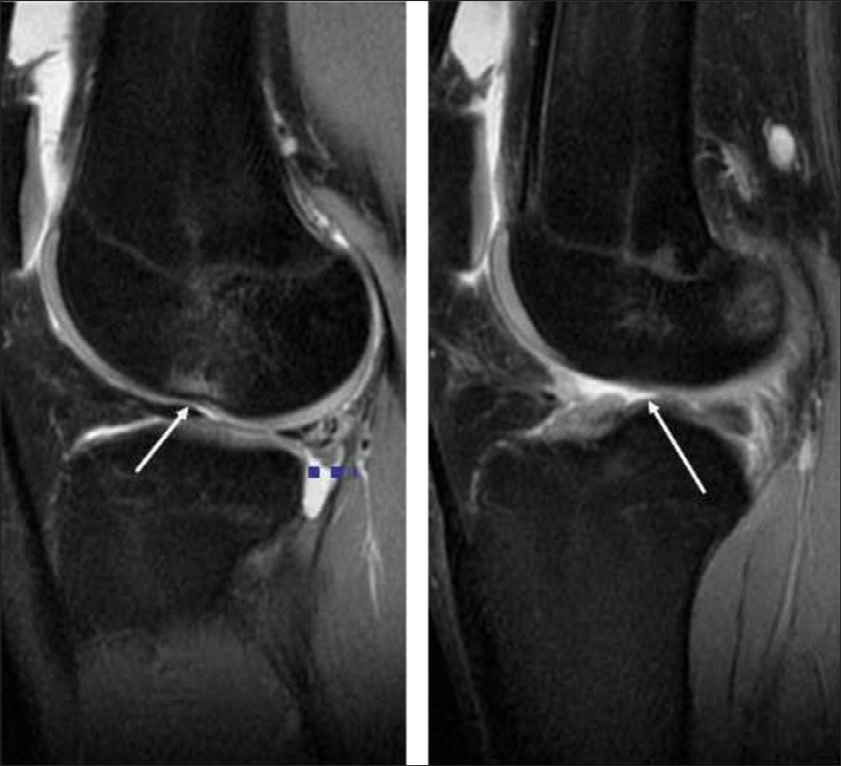
Sagittal T2-weighted MRI, typical impression in the lateral femur after a twisting injury. There is also anterior translation of the tibia compared to the femur beyond 7mm (*blue dots*). Total disruption of the ACL fibres in this patient.

**Figure 5:**
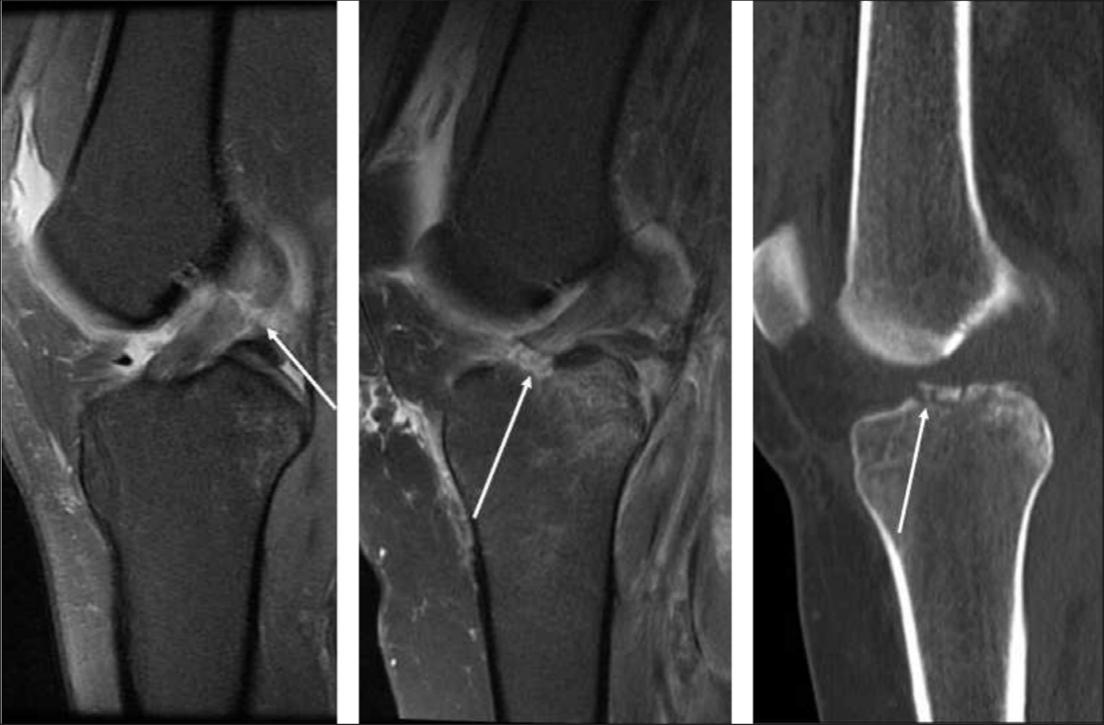
**(a)** Sagittal T2-weighted MRI, complete disruption of the ACL fibres. **(b)** Avulsion of the tibial attachment of the ACL (*arrows*). **(c)** Same patient on CT.

**Figure 6:**
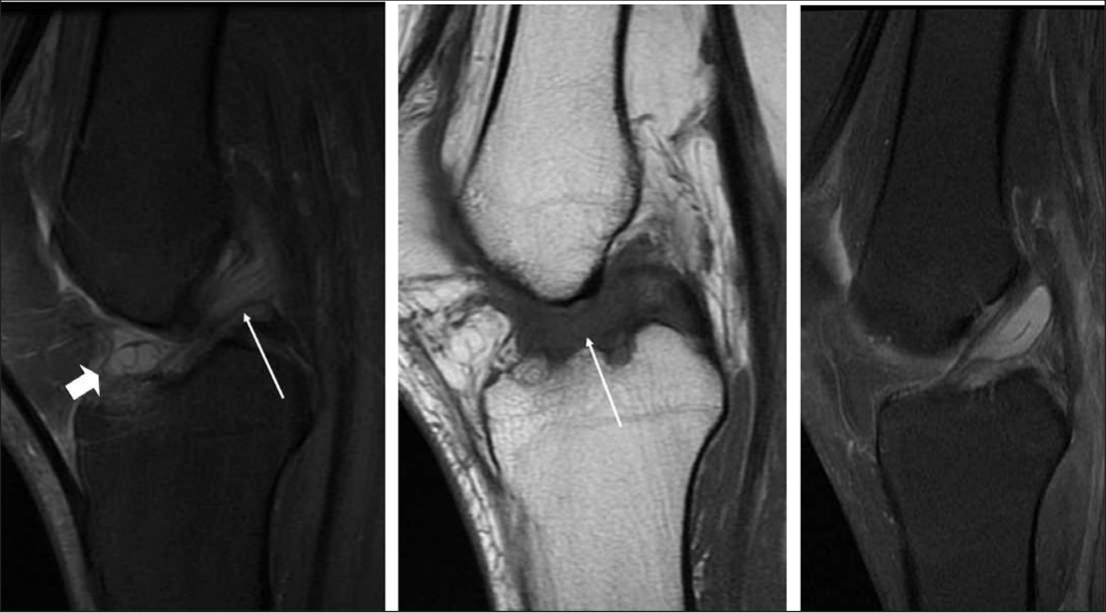
**(a)** Intraligamentous areas of increased signal on T2- and **(b)** T1-weighted MRI (*arrows*), which indicates mucoid degeneration. Ganglion cyst (a, *open arrow*). **(c)** Mucoid degeneration may later evolve into cysts as seen on sagittal T2-weithted MRI.

The ACL has low signal intensity on T1- and T2-weighted images and is seen on all three planes [5]. The ligament has a striated appearance (Figures 1a–c). Partial ruptures are defined as a disruption of part of the ligament fibres or if there is fluid signal in the ligament (Figures 2a–b). In some cases, it is difficult to decide on whether the rupture is complete (Figure 3). Additional (indirect) signs such as anterior translation of the lateral tibia and uncovering of the posterior horn of the lateral meniscus may be useful [5]. After a twisting injury, impression fracture of the lateral femur and contusion in the lateral tibia may be seen (Figures 4a–c). A total rupture is disruption of all the ligament fibres in the short axis or avulsion of either the femoral or tibial attachment (Figures 5a–c). Mucoid degeneration of the ACL is a potential pitfall that would lead to overcalling of partial ruptures if unrecognized. The clue is high signal on T2, but low to intermediate signal (not dark as when normal) on T1 images. In addition there may a celery stalk appearance, and the presence of ganglion cysts – which are associated with mucoid degeneration – which may be helpful in identification (Figures 6a–c) [6].

Surgery is often performed when the knee is unstable, with the aim to restore knee joint function and delay the onset of osteoarthritis. The surgical techniques have varied since surgery became common place in the 1980s. There have been variations in the use of graft material (synthetic, hamstrings, bone patellar tendon, achilles, quadriceps), the surgical approach (open surgery, arthroscopic transtibial or anteromedial port) or the number of grafts or tunnels (single or double bundle). In recent years anatomic reconstruction had gained in popularity, and the use of routine pre- and post-operative imaging is on the rise [7].

**Figure 7:**
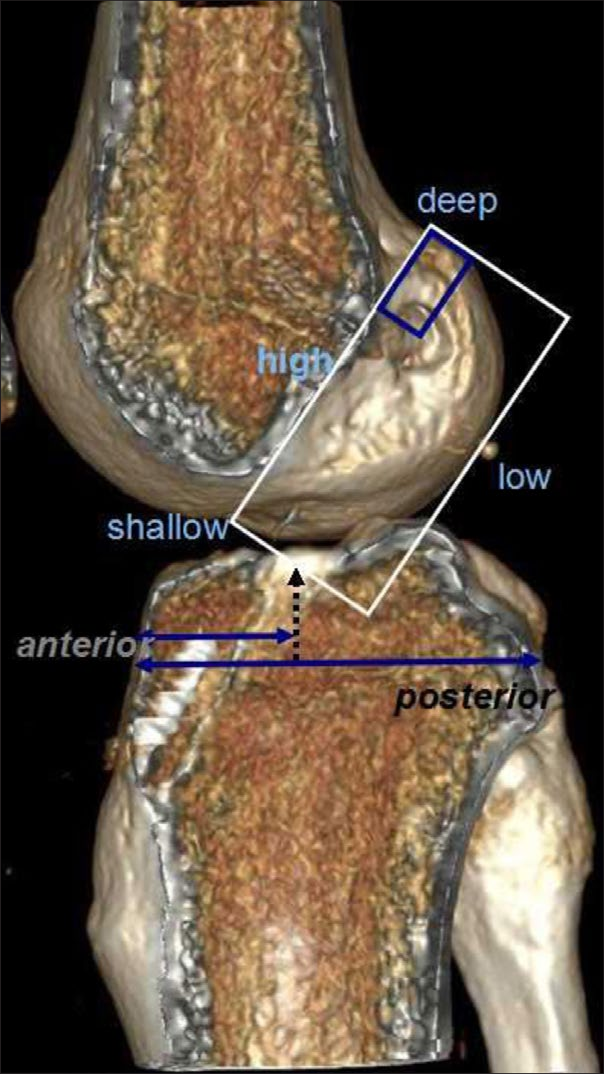
Measurements of the centres of the femoral tunnel and tibial tunnel on CT volume-rendering reconstruction. The Bernard and Hertel grid measures the femoral placement in the deep-shallow direction. The short distance is divided by the depth of the condyle. The high-low direction is the short distance divided on the height of the condyle. The tibial tunnel is the distance from the anterior border of the tibia on the entire dept of the tibia.

**Figure 8:**
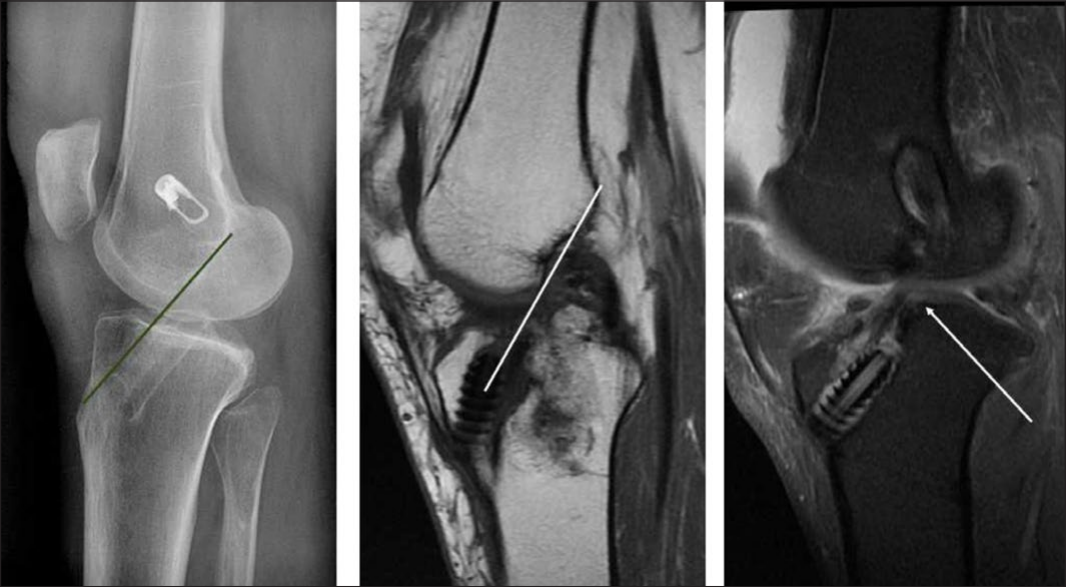
**(a)** Optimal placement of tibial tunnel, posterior to the Blumensaats line on a lateral radiograph. **(b, c)** On a different patient, part of the tibial tunnel impingement is too anterior, and the graft has a slight “s” form on sagittal T1- and T2-weighted MRI.

**Figure 9:**
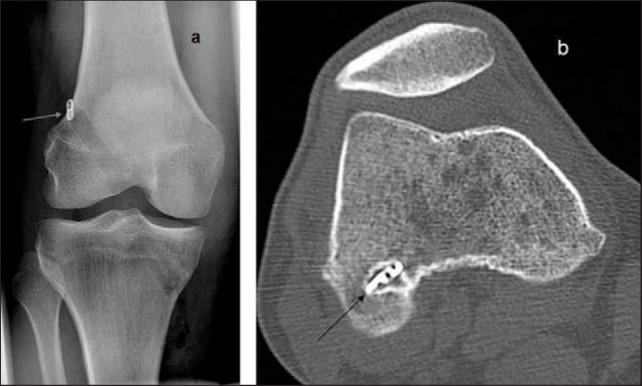
**(a)** Initially, the fixation device was barely resting on the cortex on the front radiograph. **(b)** One year later the patient returned with reduced knee function. CT showed that the device had slid into the tunnel.

Early post-operative imaging is performed to evaluate the placement of the tunnels in the femur and tibia, the tibia tunnel impingement, placement of fixation devices and as a baseline examination. The tunnels are evaluated with the Bernard and Hertel grid in the femur, and with the Stäubli and Rauschning method in the tibia in our institution (Figure 7) [8]. The tunnel placements are best evaluated with computed tomography (CT) [9]. A recent study has shown that giving the surgeons feedback about tunnel placement improves their performance [10]. The tibial impingement occurs when the tibial tunnel is not completely posterior to a line drawn from the intercondylar roof to the tibia – also called the Blumensaats line (Figure 8). It is seen when the graft has an “s” form. Sometimes magnetic resonance imaging (MRI) signal abnormalities or thinning of the fibres locally may be seen (Figure 8c). Early recognition of failed fixation devices is important to avoid later graft failure and may enable the surgeon to correct fixation devices with the same graft (Figure 9a–b) [8].

**Figure 10:**
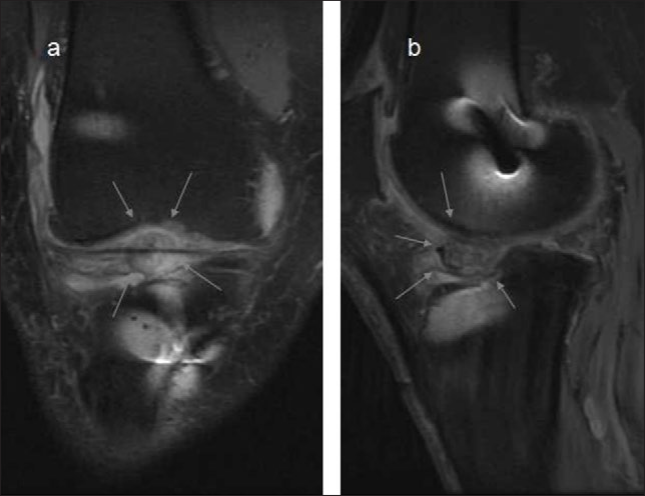
**a–b** Development of focal arthrofibrosis, soft tissue lesion with intermediate signal anterior to the graft on coronal and sagittal T2-weighted MRI (*arrows*).

**Figure 11:**
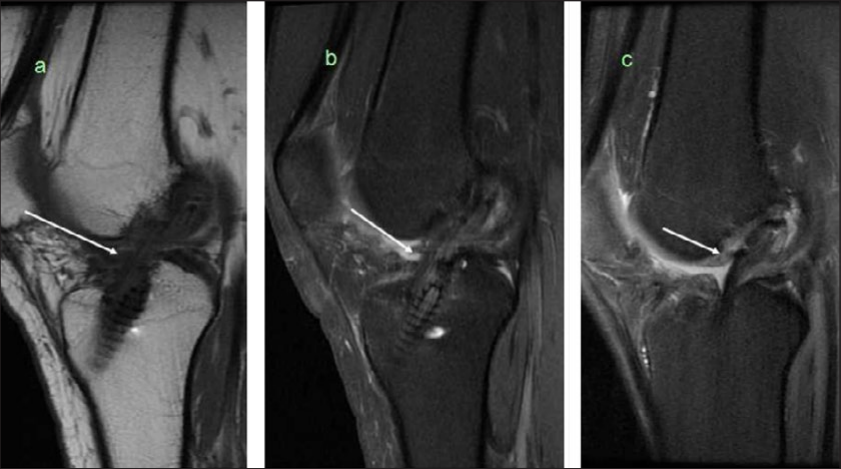
**(a)** Sagittal T1-weighted and **(b)** T2-weighted MRI showed a thickened graft with high signal at 9 months after operation. **(c)** After two years the graft is dark and resembles the native ACL.

**Figure 12:**
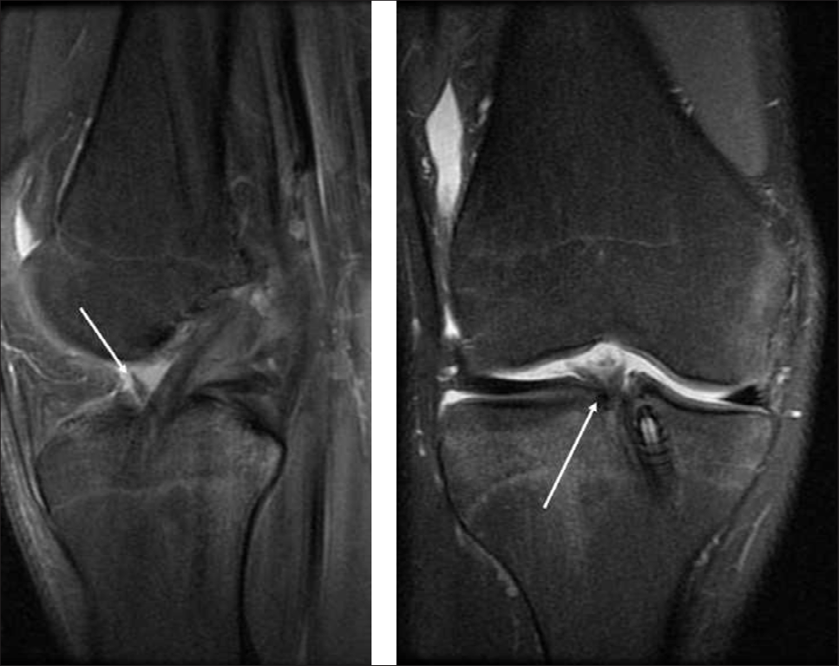
Partial rupture (verified by arthroscopy) in the anterior part of the graft (*arrows*), on sagittal and coronal T2-weighted MRI.

**Figure 13:**
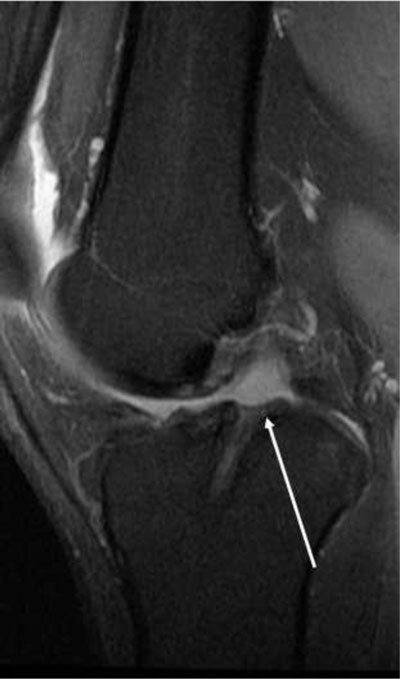
Total rupture of the graft, no visible fibres on sagittal T2-weighted MRI (*arrow*).

Later post-operative complications are tunnel widening (seen best on CT [11]), development of focal arthrofibrosis, impingement, partial or total ruptures of grafts (best appreciated on MRI) [12,13]. Focal arthofibrosis or Cyclops lesion is the development of a soft tissue lesion usually anterior to the graft, which affects knee joint function (Figure 10). Partial ruptures are diagnosed as in the native ACL, however the ligamentization process usually takes up to two years and sometime longer, so the presence of high fluid signal in the graft is not pathological in the first years (Figure 11) [14]. Indirect signs may be helpful or actual visible disruption of the fibres may be seen (Figure 12) [5]. A total rupture is like in the native ACL (Figure 13).

**Figure 14:**
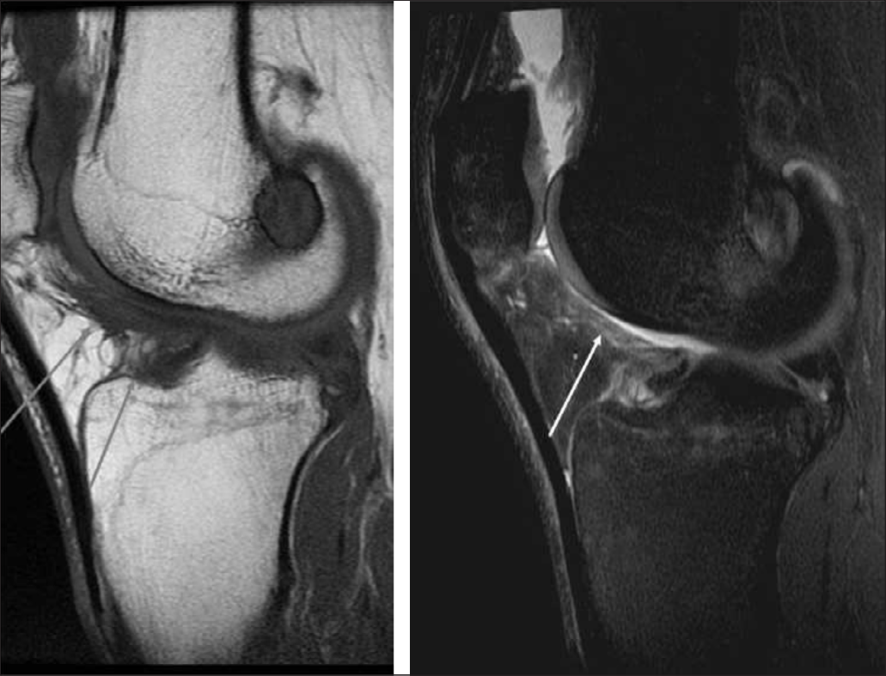
Changes in the Hoffa fat pad after surgery on sagittal T1-weighted MRI.

**Figure 15:**
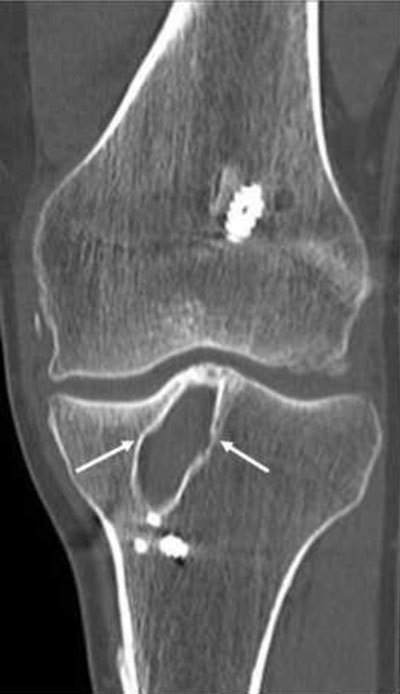
Coronal CT. Patient who had ACL construction five years ago, tunnel widening is seen in the tibia (slightly saccular expansion of the tunnel, *arrows*).

Infrapatellar fat pad disease (also called Hoffa Disease or Hoffitis) is an umbrella name for changes in the fat pad. Fat pad abnormalities are common after arthroscopy, however as not all changes cause pain or deficit in knee function, care should be taken when reporting (Figure 14) [15,16]. Considerations prior to revision surgery include tunnel placement or widening (can the same tunnels be used?) and interference of fixation devices (Figure 15) [17,18].

New trends in ACL imaging: ACL repair was a technique used in the ’70s–’80s with poor long-term outcome. In recent years ACL repair with different techniques is being performed. The early outcome studies show some promises, however a revision rate of 18% after repair in the two first years is higher than with the revision rate after reconstruction (3.6%) [19,20]. Imaging findings after these new repair techniques are still unchartered territory. ACL diffusion tension imaging (DTI) is also a new technique to assess the ligament, feasibility studies have been performed, but what additional clinical information DTI gives is yet to be determined [21].

## Posterior Cruciate Ligament (PCL) Imaging

**Figure 16:**
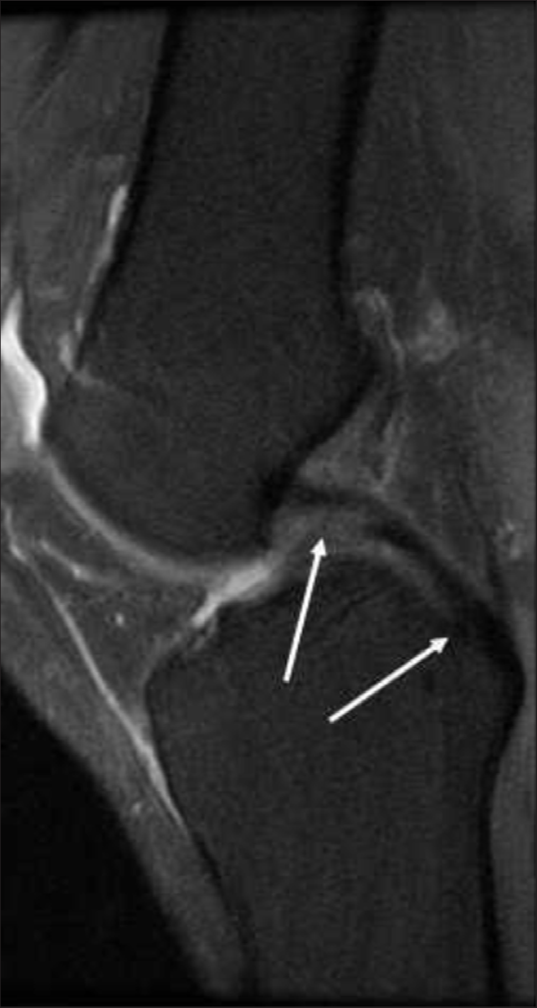
Normal signal in the PCL on sagittal T2-weighted MRI.

The PCL courses from the medial femur to the posterior part of the tibial midportion (Figure 16) [22]. It provides anterior-posterior stability, and in PCL deficient knee the medial tibia sags posteriorly. The ligament is at its longest in flexion between 90–120 degrees [23]. Common pathway for isolated PCL injuries is fall on a flexed knee or dashboard. However, isolated PCL injuries are rarer than ACL ruptures with incidence rates of 1.8/100 000 in the PCL, while the isolated ACL ruptures are 68/100 000 [24,25]. A PCL injury due to hyperextension or excessive rotation is usually associated with another ligamentous injury [26]. Avulsion of the PCL tibial insertion is seen in up to 10% of cases [27].

The normal PCL has low signal on both T1- and T2-weighted images. It appears homogenous and differs thus a little from the ACL (Figure 16) [28]. The PCL is supported by the anterior and posterior meniscofemoral ligaments [29].

**Figure 17:**
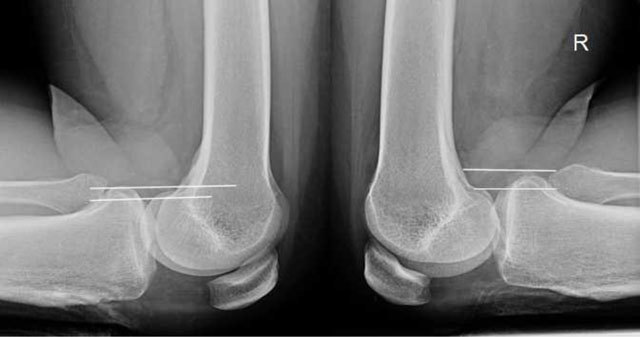
Stress radiographs of the knees, show pathological translation on the right side. It was measured to 11m whereas the normal (physiologic) translation on the left side was 5mm.

**Figure 18:**
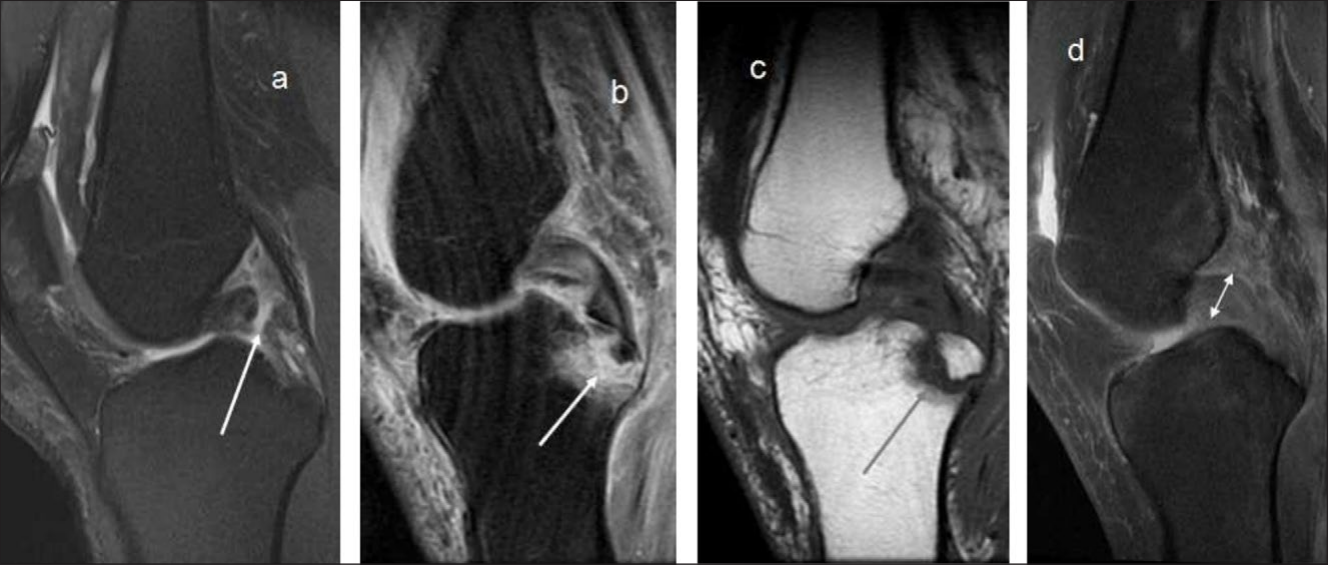
**(a)** Complete disruption of the PCL fibres. **(b, c)** Avulsion of the PCL attachment on the tibia on T2- and T1-weighted MRI (arrows). **(d)** Thick PCL >7mm, consistent with a ruptured PCL.

**Figure 19:**
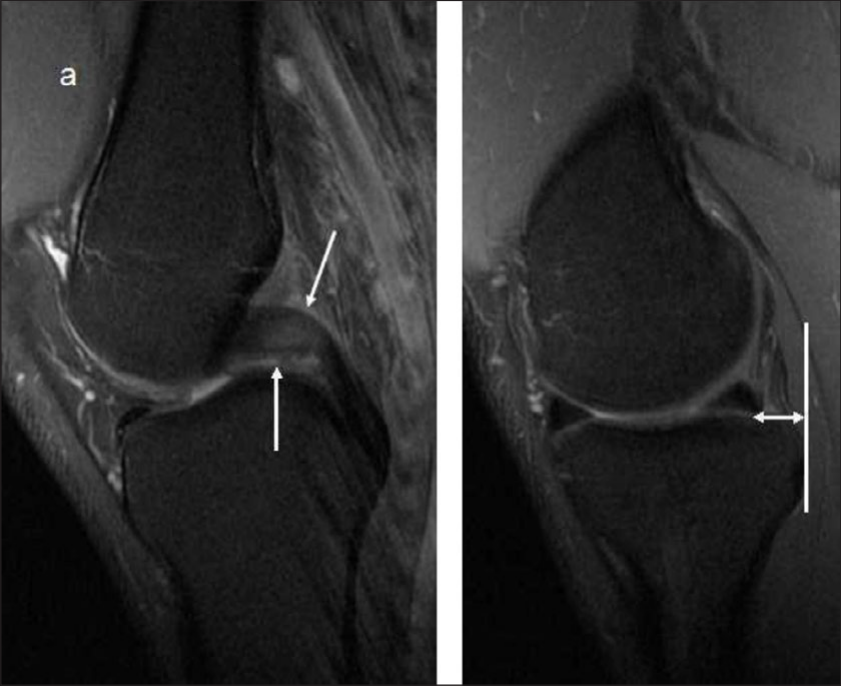
**(a)** The PCL has high signal in the proximal part, but the peripheral fibres seem intact (*arrows*), this would be called partial rupture in the ACL, but there is pathological anterior translation of the medial tibia (> 7mm) on **(b)**, indicating PCL deficiency.

A PCL deficiency causes pathological posterior sagging of the medial tibia. This is tested clinically by the surgeon, but stress radiographs are often used to objectively quantify this (Figure 17) [30]. A translation up to 7 mm is physiologic, between 8–10 mm is considered abnormal but not necessarily indication for surgical repair, while a translation above 10 mm is usually operated [31]. On MRI acute PCL injury is seen as high signal on T2-weighted images with or without disruption of the fibres, or avulsion of the attachment(s). A complete tear is also called when the PCL in the short axis is thicker than 7mm (Figure 18a–d) [32]. However, as opposed to the ACL, where intact fibres on MRI indicate an intact ACL, intact fibres in the PCL do not mean that the knee is PCL intact. The PCL may look normal and seem to have healed completely, but the knee may still be PCL-deficient (Figure 19) [33]. This is an important pitfall of MRI imaging of the PCL. Posterior translation of the medial tibia is a useful sign (Figure 19) [34]. Stress radiographs are also useful if MRI is inconclusive [35].

**Figure 20:**
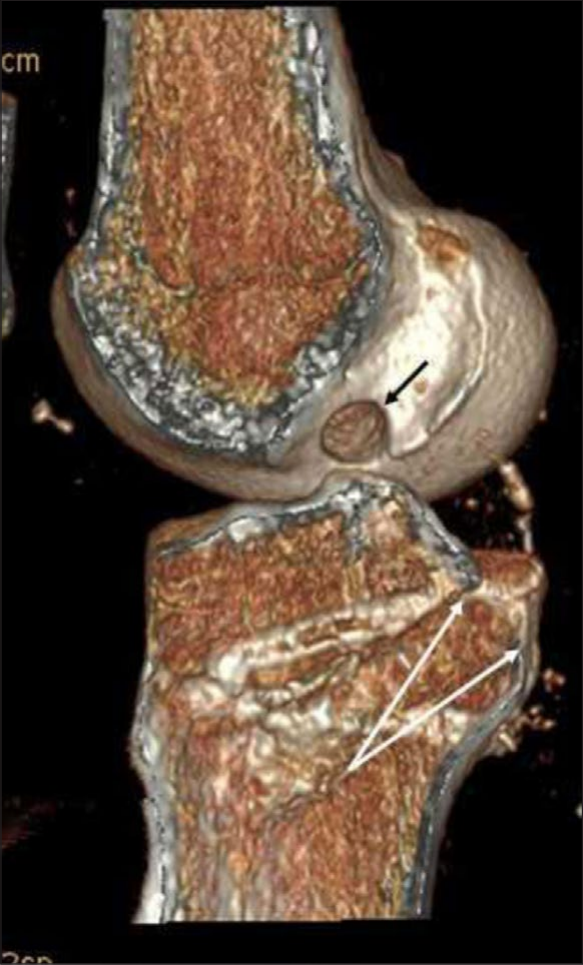
Post-operative tunnel placement after PCL reconstruction on volume rendering CT. Opening of the femoral tunnel is indicated with a *black arrow*, opening of the tibial tunnel lies between the two *white arrows*.

**Figure 21:**
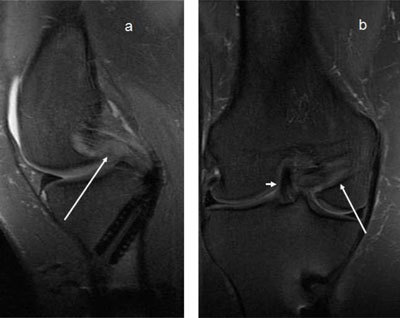
High signal in the PCL graft six months postoperatively on a sagittal and coronal T2, which is normal (*long arrows*). Short arrow shows normal ACL signal.

Compared to the ACL, the PCL surgery is not as frequently performed, but the rate of reconstruction is on the rise. The main objective is to prevent development of early osteoarthritis in the medial compartment. Surgical variations include single/double bundle, tibial inlay techniques and choice of grafts [35]. Post-operative imaging after PCL reconstruction is not performed routinely, but some do perform it for evaluation of tunnel placements and fixation devices and to serve as a baseline exam (Figure 20). On T2-weighted MRI the post-operative PCL graft shows high signal intensity, though no long-term studies have been performed on how long this remains (Figure 21). One must presume that as with the ACL graft, the same care must be taken to not overcall partial ruptures of graft based solely on graft signal. PCL revision has been reported as high as 54% [36]. Considerations for revision surgery are the same as for the ACL (i.e., tunnel placement, tunnel widening and fixation devices).

## Main Teaching Points

The MRI presentation of partial and total ruptures differs in the ACL and the PCL. High signal changes in the ACL graft may be present up to two years after surgery, and should not be called partial ruptures. Chronic PCL ruptures are easily missed on MRI. Stress radiographs may be useful in assessing PCL ruptures.
